# Give Him Air; He'll Straight Be Well

**Published:** 1902-08-23

**Authors:** 


### "Give Him Air; He'll Straight be Well."

In view of the award to Dr. Arthur Latham of the first prize, of £500, for his essay in regard to the proposed King's Sanatorium for Tuberculosis, some interest attaches to an address delivered a few months ago before the Hunterian Society of St. George's Hospital, by the successful competitor, on the Modern Treatment of Pulmonary Consumption. In it Dr. Latham throws well-deserved scorn upon the treatment which has been so often meted out to unfortunate sufferers from this disease, a treatment by-the-bye which can, even at the present day, be found in full swing in the out-patient departments of many a hospital, even of special consumption hospitals, where, if anywhere, one would expect to meet with better things. "It is not an uncommon experience," he says, "to find some unfortunate workman, who lives continuously in a fœtid atmosphere and eats an indifferent amount of coarse and unnutritious food, taking all of the following medicines during the 24 hours :—A mixture of cod-liver oil with malt, to supply, so it is said, the place of the 'fast-ebbing vital oil;' a mixture of gentian and sodium bicarbonate, to assist the jaded appetite; an ether mixture to strengthen the action of the heart when the patient feels more than usually ill; some form of lozenge to allay the cough during the day time, together with a new-fangled antiseptic as an inhalation; and some pernicious preparation of opium to bring sleep at night." It is one of our amiable weaknesses to hold patent medicines in ridicule and contempt, but what could be more ridiculous, considering the teachings of the dead-house, than the current treatment of consumption so aptly described by Dr. Latham—a mere pouring in of drugs without any attempt to touch the root of the disease. Yet in the midst of all this drugging, which has been going on far longer than we can remember, there have been men who saw the truth. So far back as 1840, George Bodington insisted on the importance of a generous diet and a constant supply of pure air, and propounded the terrible heresy that "cold is never too intense for a consumptive patient." In 1855 Dr. Henry MacCormac, the father of the late Sir William MacCormac, published a book on somewhat similar lines, and in 1861 read a paper before the Royal Medical and Chirurgical Society in which he advocated what are now established principles. Yet what was the treatment which these pioneers received at the hands of their professional colleagues? Bodington's book, says Latham, "met with much bitter and fierce opposition, and eventually the disapproval of his methods became so universal that patients were driven from his sanatorium," while "the members of the Royal Medical and Chirurgical Society refused to pass the usual vote of thanks to Dr. MacCormac, because they thought that the paper was written by a monomaniac."

The position taken up by the medical profession in regard to the treatment of consumption has indeed been most deplorable, and has thrown into strong light the bar sinister which hangs over the origin of medicine—a science, if it be a science, springing in the far past from mystery and witchcraft, tainted with the methods of the sorcerer, and even now dominated by that overmastering faith in drugs and nostrums which is the direct and disastrous heritage handed down to us by our immediate ancestors, the apothecaries. It has been an ignoble spectacle. No one taking a broad view. Each man limited by his education and trudging along in the rut of his old habits—physicians pouring in drugs, surgeons scraping out bits of diseased tissue, while even now, in the full light of bacteriological science, we find men attempting to cure consumption by soaking the patient's tissues with antiseptics; and all this in defiance of the teachings of pathology, which go to show how frequently the disease gets well if the patient's vitality, the *vis medicatrix naturæ*, is but given a fair chance. Yet, how near we were to the truth if we would but have listened, if we would but have cut ourselves adrift from the prejudices ingrained in us by our education, and, in the words of one great man, have thrown "physic to the dogs," and in those of another, have investigated all things by "observation and experiment." Once a year we have met together to do honour to the immortal Harvey, and then we have returned to this miserable drug-giving as if Harvey had never existed. Meanwhile, notwithstanding our ostracism of new ideas, the teaching of Bodington, of MacCormac, and of the modern host of sanatorium owners has prevailed; and now, at last, in the full sunshine of royal patronage, we admit how simple is the truth, expressed as it is by the motto of Dr. Latham's essay :—"Give him air; he'll straight be well." What sycophants we all are! It is high time that, as a profession, we sang a litany, "From the thraldom of dogma and the limitations of the physic bottle, Good Lord deliver us."

